# Acute kidney injury caused by ureteral obstruction after Deflux^®^ injection treatment for vesicoureteral reflux after pediatric kidney transplantation: a case report

**DOI:** 10.1186/s12894-025-01733-7

**Published:** 2025-03-14

**Authors:** Yujiro Aoki, Yuko Hamasaki, Junya Hashimoto, Ayuko Zaitsu, Maho Maeda, Masaki Muramatsu, Takeshi Kawamura, Seiichiro Shishido, Ken Sakai

**Affiliations:** Department of Nephrology, Toho University Faculty of Medicine, 6-11-1 Omori-nishi, Ota-ku, Tokyo, 143-8541 Japan

**Keywords:** Pediatric, Kidney transplantation, Urinary tract obstruction, Deflux^®^, Acute kidney injury, Vesicoureteral reflux

## Abstract

**Background:**

Vesicoureteral reflux (VUR) after pediatric kidney transplantation (KT) is a frequent urologic complication. Endoscopic Deflux^®^ injection is a treatment option. However, ureteral obstruction after Deflux^®^ injection treatment is a potential complication that may have serious outcomes for patients who have undergone KT. We report a case of acute kidney injury (AKI) caused by ureteral obstruction with acute foreign body reaction immediately after Deflux^®^ injection treatment for VUR after transplantation.

**Case presentation:**

We encountered a 5-year-old boy who underwent living-donor KT at age 4 years because of end-stage kidney disease caused by posterior urethral valves. At 4 months after KT, VUR (grade IV) and bladder dysfunction worsening were detected by voiding cystourethrography. A second febrile urinary tract infection (UTI) was treated with endoscopic Deflux^®^ injection in the neo-orifice of the transplanted kidney after 2 weeks of antimicrobial therapy. Postoperatively, the patient experienced a temporary decrease in urine output. Increased creatinine was observed on postoperative day 1. The renal pelvis was more dilated than it was preoperatively, and ureteral dilatation was observed. A bulge associated with Deflux^®^ injection, consistent with the injection site, was observed in the bladder. Additionally, because the graft function continued to decline, AKI associated with ureteral obstruction after Deflux^®^ injection treatment was diagnosed, and a ureteral stent was placed on postoperative day 4. The graft function gradually recovered. Four months later, the ureteral stent was removed. Exacerbation of hydronephrosis of the transplanted kidney was not observed, and the graft function was stable. Although the patient experienced residual VUR after KT, excretion control was continued and UTI recurrence was not observed.

**Conclusions:**

Ureteral obstruction after Deflux^®^ injection treatment for VUR after transplantation is a serious complication; therefore, treatment indications and timing should be carefully considered.

## Background

Vesicoureteral reflux (VUR) after pediatric kidney transplantation (KT) is a frequent urological complication that occurs in 19–60% of patients who have undergone an anti-reflux procedure [1–4]. VUR of the transplanted kidney is usually not a problem in the absence of urinary tract infections (UTIs); however, surgical intervention is considered for VUR associated with recurrent febrile UTIs [5]. Dextranomer/hyaluronic acid (Deflux^®^; Q-Med AB, Uppsala, Sweden) has been widely used for the treatment of VUR, and endoscopic Deflux^®^ injection treatment is an established therapy for VUR in the native kidney [6, 7]. However, Deflux^®^ injection treatment for VUR after transplantation has not resulted in consistent outcomes [8–13] and is associated with an average success rate of only 36.8% [14]. Additionally, ureteral obstruction—a complication that can occur after Deflux^®^ injection treatment—leads not only to decreased graft function but also difficulty performing open ureteroneocystostomy as a salvage procedure for obstruction after Deflux^®^ injection treatment [12, 13]. Ureteral obstruction after Deflux^®^ injection treatment for VUR after transplantation has been observed in 10.4% of patients, with various reports of early and late onset [14]. We report our experience with a case of VUR after transplantation who developed acute kidney injury (AKI) with acute foreign body reaction immediately after Deflux^®^ injection treatment.

## Case presentation

**Fig. 1:**
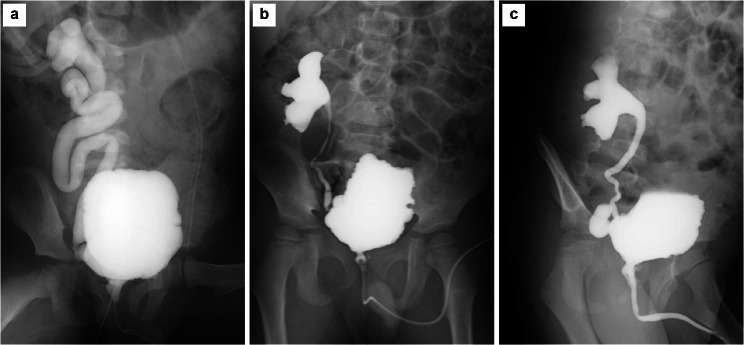
Voiding cystourethrography (VCUG) findings before and 4 months after kidney transplantation. (**a**) VCUG before transplantation showing severe bladder trabeculation and grade V right vesicoureteral reflux (VUR). The maximum cystometric capacity (MCC) is 150 mL. In the voiding phase, there is no urethral stricture. The patient is able to void without residual urine. (**b** and **c**) VCUG findings at 4 months after transplantation showing VUR (grade IV) after transplantation and VUR in the residual ureter of the right native kidney. VUR to the transplanted kidney early during the filling phase, bladder wall irregularity, and severe bladder trabeculation are observed (**b**). Dilation of the ureter is observed during the voiding phase (MCC data unknown) (**c**)

**Fig. 2:**
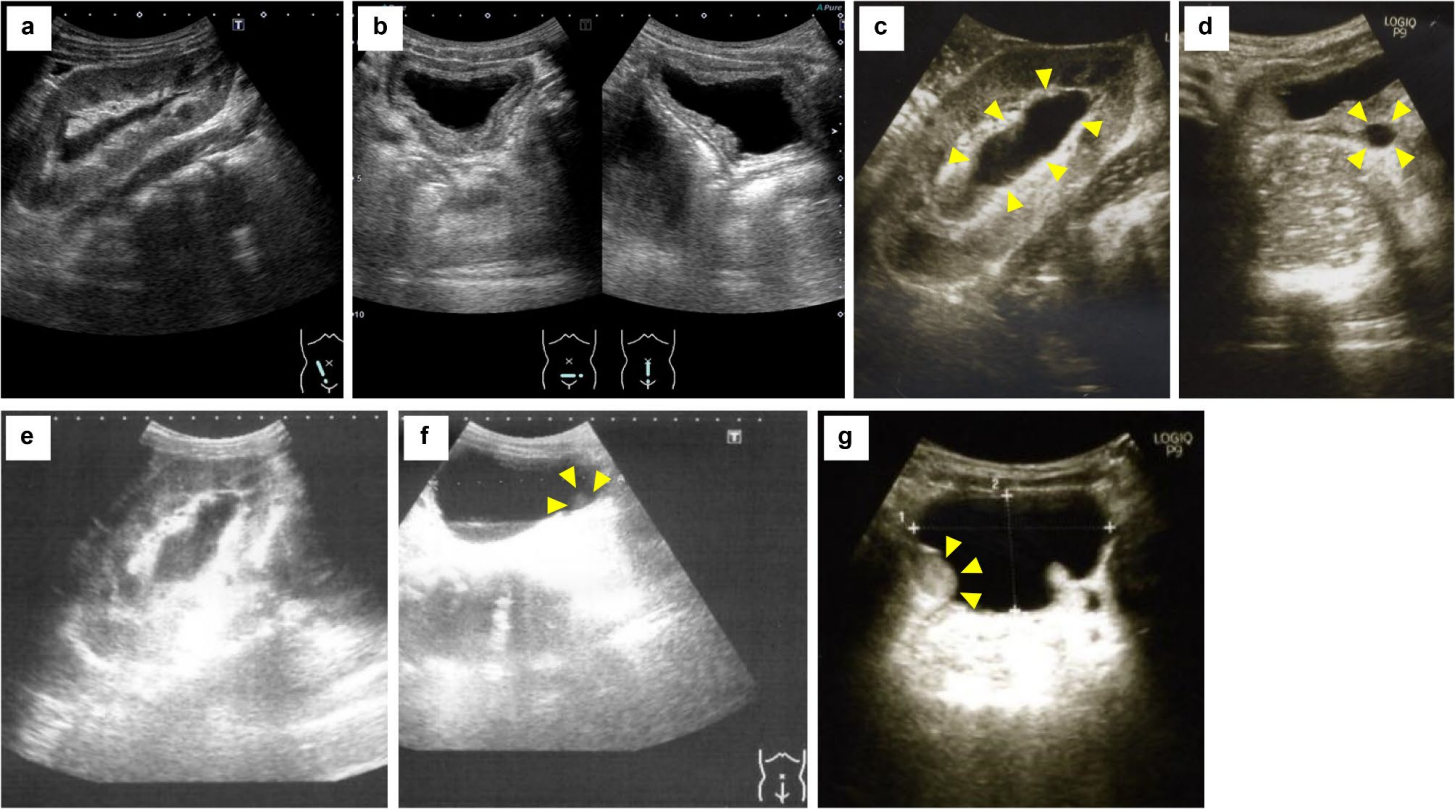
Ultrasonography findings at 4 months after kidney transplantation (KT) and after Deflux^®^ injection treatment. At 4 months after KT, ultrasonography shows mild hydronephrosis of the transplanted kidney (Society of Fetal Urology [SFU] grade 1) (**a**). No dilation of the distal ureter is observed, and the bladder wall is thickened (**b**). Ultrasonography immediately after surgery shows that the renal pelvis is more dilated than it was preoperatively (**c**). Ureteral dilatation is observed (yellow arrowhead) (**d**). (**e** and **f**) Postoperative day 1. After Deflux^®^ injection treatment, hydronephrosis of the transplanted kidney remains unchanged (SFU grade 1) (**e**). A bulge associated with Deflux^®^ is observed at a location consistent with the injection site (yellow arrowhead) (**f**). Postoperative day 3. After Deflux^®^ injection treatment, a bulge associated with Deflux^®^ at a location consistent with the injection site is observed (yellow arrowhead) (**g**)

**Fig. 3:**
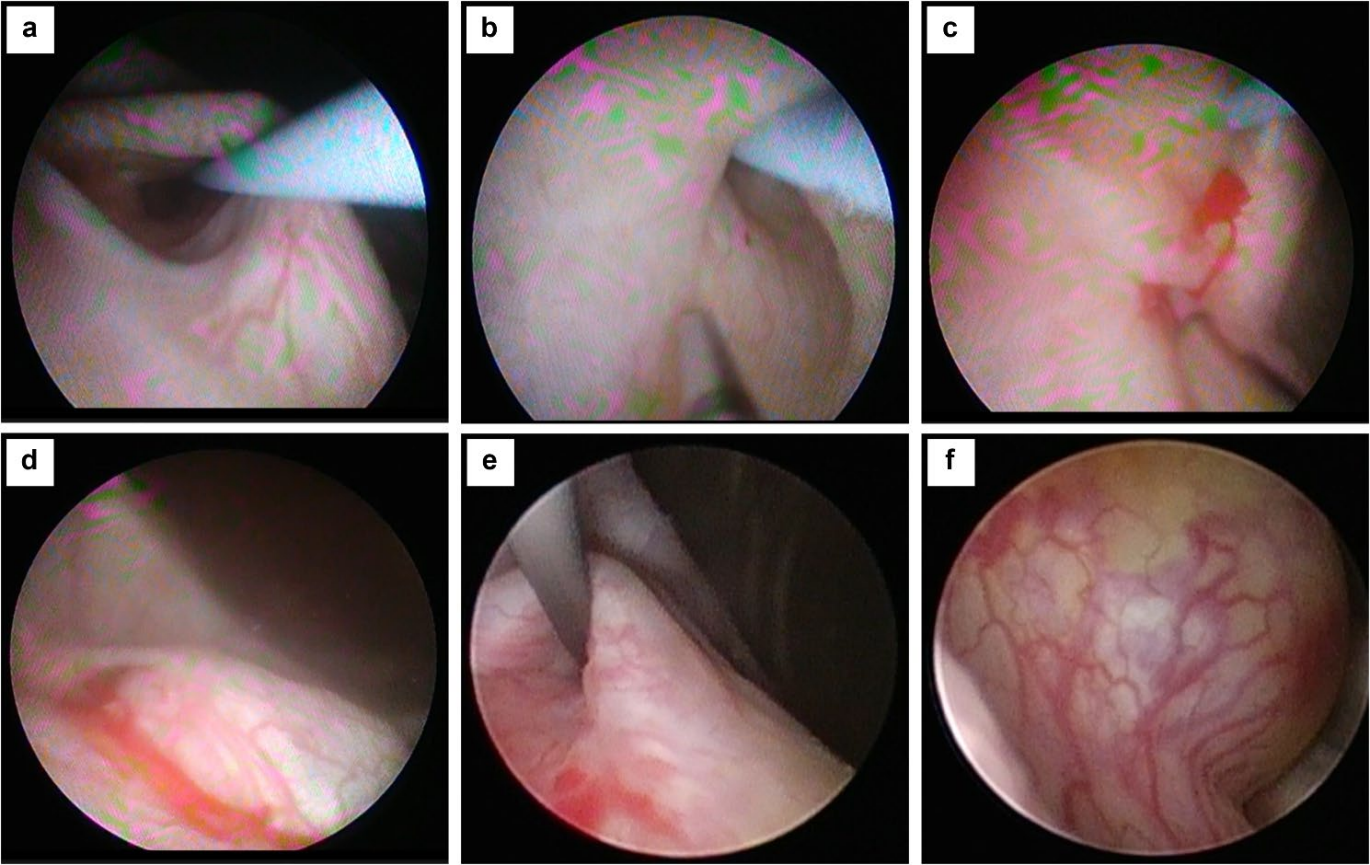
Cystoscopy findings before and after Deflux^®^ injection treatment. (**a**) The neo-orifice of the transplanted kidney is identified at the bladder sidewall on the outer side of the right ureteral orifice. Cystoscopy shows moderate trabeculation in the bladder. A 0.035-inch-diameter hybrid guidewire (Sensor™ Straight Tip; Boston Scientific) is inserted in the neo-orifice of the transplanted kidney. The neo-orifice is easily observed during the guidewire procedure (**b**). Deflux^®^ is injected in the neo-orifice using the hydrodistension implantation technique (HIT) (**c**) and subureteral transurethral injection (STING) procedure (**d**). The Deflux^®^ injection volume was 1.0 mL for the HIT and 0.7 mL for the STING procedure. After Deflux^®^ injection, the open neo-orifice became a slit-like orifice with a bulge associated with Deflux^®^ (d). Four days after Deflux^®^ injection treatment, the guidewire is easily inserted in the neo-orifice of the transplanted kidney (**e** and **f**). Cystoscopy shows an increase in the bulge associated with Deflux^®^ compared to that observed immediately after injection (**g**)

A 5-year-old boy who underwent KT experienced AKI after Deflux^®^ injection treatment for VUR following transplantation. An emergency ureteral stent was placed because ureteral obstruction occurred. The patient had been on peritoneal dialysis since the age of 4 months because of end-stage kidney disease associated with posterior urethral valves (PUVs). At age 3 months, he had undergone an endoscopic transurethral incision for PUVs at the referring hospital and had spontaneous voiding with oxybutynin hydrochloride (0.13 mg/kg). The patient presented to our hospital for KT. Preoperative voiding cystourethrography showed severe bladder trabeculation and grade V right VUR (Fig. 1a). The patient was able to store up to 150 mL of urine, had no urethral stricture, and was able to void without residual urine. A video urodynamic study showed low bladder compliance (10 mL/cmH_2_O), and no evidence of high pressure in the detrusor during voiding was observed. Preoperative management of bladder dysfunction involved oxybutynin hydrochloride and timed voiding. The patient underwent ABO-incompatible KT (blood type of A to O) at age 4 years with his father as the donor. Surgery was performed using the right retroperitoneal cavity as the graft bed. KT was performed after prior removal of the right kidney with high-grade VUR ureteral implantation performed using the extravesical approach and the Lich-Gregoir method. A 5-Fr ureteral stent was inserted and then removed on postoperative day 5. Subsequently, mild hydronephrosis (Society of Fetal Urology grade 1) and an increased creatinine level (from 0.5 mg/dL to 0.9 mg/dL) were observed. On postoperative day 7, we attempted cystoscopy and retrograde pyelography under general anesthesia but were unable to insert a guidewire and ureteral catheter into the ureteral opening of the transplanted kidney. Therefore, we performed an anterograde pyelogram after puncture of the dilated renal pelvis of the transplanted kidney.Antegrade pyelography showed that the contrast flow from the renal pelvis to the bladder was good and that the creatinine level decreased. Sulfamethoxazole/trimethoprim prophylaxis (1.2 g/day) was initiated postoperatively, and the patient was discharged without oxybutynin hydrochloride. At 4 months after KT, VUR after transplantation (grade IV) and VUR in the residual ureter of the right native kidney were detected by voiding cystourethrography (Fig. 1b, c). VUR to the transplanted kidney early during the filling phase, bladder wall irregularity, and severe bladder trabeculation were observed. VUR in the residual ureter of the right native kidney was observed during the voiding phase. Ultrasonography showed mild hydronephrosis (Society of Fetal Urology grade 1) of the transplanted kidney (Fig. 2a, b). The patient experienced daytime urinary incontinence and nocturia. Based on the voiding cystourethrography results, the bladder function was considered worse postoperatively; therefore, oxybutynin hydrochloride (0.3 mg/kg) was initiated and sulfamethoxazole/trimethoprim prophylaxis was continued. A febrile UTI with bacteremia caused by extended-spectrum β-lactamase-producing *Klebsiella pneumoniae* developed. Subsequently, a second febrile UTI developed. Therefore, after 2 weeks of antimicrobial therapy, the patient was promptly administered endoscopic Deflux^®^ injection treatment for VUR after KT. The bladder was observed using a rigid 30° cystoscope. The neo-orifice of the transplanted kidney was identified at the bladder sidewall, on the outer side of the right ureteral orifice. However, Deflux^®^ injection proved challenging. Therefore, a 0.035-inch-diameter hybrid guidewire (Sensor™ Straight Tip; Boston Scientific) was inserted in the neo-orifice of the transplanted kidney, lifted, and manipulated to achieve visibility of the ureteral opening. A total of 1.7 mL of Deflux^®^ was injected using the hydrodistension implantation technique (1.0-mL injection) [15] and subureteral transurethral injection procedure (0.7-mL injection) [16] with a guidewire inserted in the neo-orifice of the transplanted kidney (Fig. 3a–d). The VUR of the left native kidney had not been previously delineated, although preoperative ultrasound showed ureteral dilatation. In addition, cystoscopy prior to Deflux^®^ injection showed that the ureteral orifice of the left native kidney was type H1 (dynamic hydrostatic dilation classification system). Therefore, 1.3 mL of Deflux^®^ was injected into the left ureteral orifice using the hydrodistension implantation technique to prevent urinary tract infection. Postoperatively, the patient experienced a temporary decrease in urine output, painful urination and hematuria, and an increased creatinine level (from 0.69 mg/dL to 1.62 mg/dL) on postoperative day 1. The renal pelvis was more dilated than it was preoperatively, and ureteral dilatation was observed (Fig. 2c–e). A bulge associated with Deflux^®^ in the bladder consistent with the injection site was observed (Fig. 2f, g). Additionally, the creatinine level increased to 3.11 mg/dL and the graft function continued to decline. The patient’s bladder dysfunction might have caused a rapid foreign body reaction to the Deflux^®^ injection immediately after treatment for UTI. Furthermore, the injection for Deflux^®^ treatment was performed outside the anatomical target — at the neo-apical orifice of the transplanted kidney — making it difficult to inject the drug into the appropriate site. Therefore, AKI associated with ureteral obstruction after Deflux^®^ injection treatment was diagnosed, and a 4.8-Fr ureteral stent with a length of 14 cm (Tria™; Boston Scientific, Marlborough, MA, USA) was placed on postoperative day 4 (Fig. 3e, f). Increased urinary outflow occurred after ureteral stent placement, the creatinine level decreased to 1.04 mg/dL, and the graft function gradually recovered. Four months later, the ureteral stent was removed. Exacerbation of hydronephrosis of the transplanted kidney was not observed and the graft function was stable. Although the patient experienced residual VUR after KT, excretion control was continued and UTI did not recur.

## Discussion and conclusions

Approximately 40% of pediatric kidney transplant recipients experience UTIs [17]. In particular, VUR after transplantation is a known risk factor for recurrent febrile UTIs [1, 2, 18], and recurrent pyelonephritis can lead to graft loss [19]. Patients who experience recurrent febrile UTIs associated with VUR after transplantation require surgical treatment. Since the Food and Drug Administration approved Deflux^®^ in 2001, it has become the most common endoscopic bulking agent used for the treatment of VUR [7]. Endoscopic Deflux^®^ injection treatment is less invasive than open surgery and is recognized as a common initial treatment that can improve VUR after KT [5].

The success rates of Deflux^®^ injection treatment for pediatric VUR after transplantation range from 0 to 63.6% [8–13]. Compared with the success rates of up to 90% for primary VUR in pediatric patients [15], the success rates of Deflux^®^ injection treatment for pediatric VUR after transplantation are much lower. Therefore, in addition to the experience of the surgeon and technical issues, scarring at the ureteral anastomosis site and the anatomic location of the transplanted ureter may lead to difficulty with needle access and proper injection, thus preventing consistent outcomes [14]. Additionally, inadequately treated bladder dysfunction can influence the success rates of endoscopic therapy [20]. With primary pediatric VUR, the risk of persistent VUR after Deflux^®^ injection treatment for children with bladder bowel dysfunction is high; although bladder bowel dysfunction is not a contraindication to endoscopic treatment, it should be treated before surgical intervention for VUR [21]. The incidence of ureteral obstruction after Deflux^®^ injection treatment for primary VUR is between 0.5% and 6.1% [22]. However, the incidence of ureteral obstruction after Deflux^®^ injection treatment for pediatric VUR after transplantation is proportionally higher: 10.4% [14]. The onset of ureteral obstruction can be broadly classified as early onset, when it occurs within a few days after surgery, or delayed onset, when it occurs several weeks or more after surgery. Patients with early-onset ureteral obstruction have an acute presentation comprising pain, nausea, vomiting, or anuria within several days of injection [22]. Acute cases of ureteral obstruction may occur because of increased resistance at the ureterovesical junction caused by the tissue augmenting substance itself or injection in the wrong tissue plane [22]. Delayed-onset ureteral obstruction, however, may be discovered incidentally with asymptomatic hydroureteronephrosis and requires regular long-term follow-up [22–24]. Furthermore, delayed-onset ureteral obstruction is associated with increased injections because of a foreign body reaction, ineffective ureteral peristalsis, adhesions, or acute/chronic inflammation [22, 24]. Arlen et al. reported that a higher volume of injected drugs is removed during open surgery after Deflux^®^ injection treatment compared to that injected at the time of treatment, indicating that it is strongly influenced by foreign body reactions after injection [24]. Our patient with PUVs had recurrent febrile UTI due to inadequate management of bladder dysfunction after KT. Deflux^®^ injection was administered immediately after UTI treatment. Immediately after surgery, the patient exhibited decreased urine output. Ultrasonography confirmed transplanted kidney hydronephrosis with acute-onset ureteral obstruction. One day after surgery, the bulge associated with Deflux^®^ in the bladder expanded rapidly. When bacteria adhere to the bladder mucosa causing a urinary tract infection, innate immunity is activated, inflammatory cytokines are released, neutrophils and macrophages gather on the bladder mucosa, vascular permeability is increased, and the inflammatory state persists [25]. In addition, Chertin et al. examined predictors of ureteral obstruction in endoscopic surgery for native kidney VUR in children, and univariate analysis revealed that grade V reflux, presence of the pretreatment beak sign studied, and inflammation of the bladder mucosa during injection were important independent risk factors leading to ureteral obstruction [26]. Therefore, the ureteral obstruction and the rapid increase in Deflux^®^ in the bladder could have been due to a foreign body reaction more intense than usual because the Deflux^®^ injection was performed immediately after UTI treatment with ongoing inflammation of the bladder mucosa.

In children with PUVs, myogenic changes in the bladder wall cause abnormal voiding behavior and increased pressure in the bladder, thus leading to fibrosis in the bladder wall [27]. Our patient had undergone KT for end-stage kidney disease caused by PUVs and experienced bladder wall thickening. Furthermore, during the ureteral implantation procedure, the neo-orifice of the transplanted kidney was created in a non-anatomically distinct location. Therefore, difficulty injecting Deflux^®^ in the proper site was possible; furthermore, Deflux^®^ can stray into areas other than the submucosa of the bladder. Cambareri et al. reported four cases of ureteral obstruction after Deflux^®^ injection treatment for VUR after transplantation. All patients were injected with Deflux^®^ using the subureteral transurethral injection method. Three of these patients had early-onset ureteral obstruction, and two of these patients had bladder dysfunction. The ureteral stent was placed promptly. For one patient, the ureteral stent was removed after 4 weeks without problems; however, the ureteral obstruction was not released in two patients, and one patient underwent open ureteroneocystostomy [12]. Ureteral obstruction of the transplanted kidney leads to rapid renal function deterioration. We suspected acute ureteral obstruction after Deflux^®^ injection and monitored transplant renal function and dilation of the renal pelvis ureter daily. Especially in children, general anesthesia is required for cystoscopy and ureteral stent insertion, and the risk associated with anesthesia may increase as the condition worsens. Therefore, close monitoring must be kept in mind so that the timing of ureteral stent placement is not missed. Rebullar et al. found that open ureteral reimplantation was performed for four (57%) of seven patients with ureteral obstruction after Deflux^®^ injection treatment for VUR after transplantation, and two (29%) of these seven patients experienced successful treatment outcomes [14]. Therefore, open ureteral reimplantation after Deflux^®^ injection treatment is challenging [12, 13]. Although the risk factors for ureteral obstruction after Deflux^®^ injection treatment of VUR after transplantation are unknown, this case suggests that a rapid foreign body reaction caused by Deflux^®^ injection after early UTI treatment occurred and was influenced by inadequate management of bladder dysfunction and failure to inject Deflux^®^ at the appropriate site in the neo-orifice of the transplanted kidney because of its non-anatomic location.

In conclusion, VUR to the transplanted kidney may require intervention if loss of graft function or recurrent UTIs occur. Although Deflux^®^ injection treatment may be beneficial for pediatric patients who require intervention for VUR, ureteral obstruction is a potential complication after Deflux^®^ injection treatment that has serious consequences because it is directly related to loss of graft function. Clinicians should be aware that ureteral obstruction attributable to Deflux^®^ injection treatment for VUR after transplantation is a serious potential complication. Particularly, treatment of VUR in children with bladder dysfunction, appropriate excretion control, and treatment indications and timing should be carefully considered.

## Data Availability

The datasets used and analyzed during the current study are available from the corresponding author upon reasonable request.

## Abbreviations

AKI: Acute kidney injury
KT: Kidney transplantation
PUV: Posterior urethral valve
UTI: Urinary tract infection
VUR: Vesicoureteral reflux
